# Cerebrospinal fluid *Plasmodium falciparum* histidine-rich protein-2 in pediatric cerebral malaria

**DOI:** 10.1186/s12936-018-2272-y

**Published:** 2018-03-23

**Authors:** Kiran T. Thakur, Jimmy Vareta, Kathryn A. Carson, Samuel Kampondeni, Michael J. Potchen, Gretchen L. Birbeck, Ian MacCormick, Terrie Taylor, David J. Sullivan, Karl B. Seydel

**Affiliations:** 1 Division of Critical Care and Hospitalist Neurology, Department of Neurology, Columbia University Medical Center, 177 Fort Washington Avenue, Milstein Hospital, 8GS-300, New York, NY 10032 USA; 2 Blantyre Malaria Project, University of Malawi College of Medicine, Blantyre, Malawi; 3 Department of Epidemiology, Johns Hopkins Bloomberg School of Public Health, Baltimore, MD USA; 4 Neuroradiology Division, Department of Imaging Sciences, University of Rochester, Rochester, NY USA; 5 Queen Elizabeth Central Hospital, Blantyre, Malawi; 6 Epilepsy Division, Department of Neurology, University of Rochester Medical Center, Rochester, NY USA; 7 Malawi-Liverpool-Wellcome Trust Clinical Research Programme, Blantyre, Malawi; 8 Department of Eye and Vision Science, University of Liverpool, Liverpool, UK; 9 Centre for Clinical Brain Sciences, University of Edinburgh, Edinburgh, UK; 10 Department of Osteopathic Medical Specialties, College of Osteopathic Medicine, Michigan State University, East Lansing, MI USA; 11 W. Harry Feinstone Department of Molecular Microbiology and Immunology, Johns Hopkins Bloomberg School of Public Health, Baltimore, MD USA

**Keywords:** Coma, Parasitic infections, Cerebrospinal fluid

## Abstract

**Background:**

Cerebral malaria (CM) causes a rapidly developing coma, and remains a major contributor to morbidity and mortality in malaria-endemic regions. This study sought to determine the relationship between cerebrospinal fluid (CSF) *Plasmodium falciparum* histidine rich protein-2 (PfHRP-2) and clinical, laboratory and radiographic features in a cohort of children with retinopathy-positive CM.

**Methods:**

Patients included in the study were admitted (2009–2013) to the Pediatric Research Ward (Queen Elizabeth Central Hospital, Blantyre, Malawi) meeting World Health Organization criteria for CM with findings of malarial retinopathy. Enzyme-linked immunosorbent assay was used to determine plasma and CSF PfHRP-2 levels. Wilcoxon rank-sum tests and multivariable logistic regression analysis assessed the association of clinical and radiographic characteristics with the primary outcome of death during hospitalization.

**Results:**

In this cohort of 94 patients, median age was 44 (interquartile range 29–62) months, 53 (56.4%) patients were male, 6 (7%) were HIV-infected, and 10 (11%) died during hospitalization. Elevated concentrations of plasma lactate (p = 0.005) and CSF PfHRP-2 (p = 0.04) were significantly associated with death. On multivariable analysis, higher PfHRP-2 in the CSF was associated with death (odds ratio 9.00, 95% confidence interval 1.44–56.42) while plasma PfHRP-2 was not (odds ratio 2.05, 95% confidence interval 0.45–9.35).

**Conclusions:**

Elevation of CSF, but not plasma PfHRP-2, is associated with death in this paediatric CM cohort. PfHRP-2 egress into the CSF may represent alteration of blood brain barrier permeability related to the sequestration of parasitized erythrocytes in the cerebral microvasculature.

**Electronic supplementary material:**

The online version of this article (10.1186/s12936-018-2272-y) contains supplementary material, which is available to authorized users.

## Background

Despite ongoing efforts to implement existing control strategies and develop candidate vaccines, severe malaria continues to generate a heavy burden of illness worldwide. In 2014, there were approximately 212 million malaria cases and 429,000 malaria deaths globally, 70% occurring in children under the age of 5 years living in sub-Saharan Africa [1]. Cerebral malaria (CM), the deadliest form of the disease, with a mortality rate of approximately 15–25%, is defined as a Blantyre Coma Score (BCS) of 2 or less, peripheral *Plasmodium falciparum* parasitaemia and no other discernible cause of coma [2]. Additionally, retinal signs have been found to be over 95% sensitive and specific for pre-morbid identification of children with significant central nervous system (CNS) parasite sequestration at autopsy [3–5].

Histopathologically, CM is characterized by sequestration of mature parasites in the microvasculature of the brain [6–8]. Sequestration results from the expression of parasite proteins on the surface of the infected erythrocyte, which mediates the attachment of the parasitized erythrocyte to endothelial cells of the microvasculature. At sites of sequestration, parasites continue to mature and finally rupture from host erythrocytes to release their progeny. Sequestration is thought to cause microvascular obstruction with consequent metabolic and inflammatory derangements [9, 10]. Disease progression and death in some children with CM is likely secondary to extravascular parenchymal changes driven by intravascular pathology. In an autopsy study of paediatric patients with CM, diffuse axonal and myelin damage in the brain parenchyma was present in cases where there was prominent infected erythrocyte sequestration [11]. Additional pathological findings include concentrated intravascular haemozoin in regions of sequestration, and evidence of blood brain barrier (BBB) breakdown, associated with perivascular ring haemorrhages distributed in the subcortical white matter, corpus callosum, basal ganglia and the cerebellum [12]. Neuroimaging studies have shown evidence of various patterns of parenchymal damage in CM patients, involving the basal ganglia, supratentorial white matter, brainstem and cerebral cortex [13–16]. Brain magnetic resonance imaging (MRI) in CM children shows that increased brain volume is associated with death [17]. Parasites are not seen within the brain parenchyma, and the cause of changes that lead to brain swelling, with neurological damage and death in some patients, is incompletely understood. Identification of *Plasmodium*-specific biological markers in the cerebrospinal fluid (CSF) may further elucidate mechanisms underlying the pathogenesis of CM.

*Plasmodium falciparum* histidine rich protein-2 (PfHRP-2), the basis of many rapid diagnostic tests for malaria, is a water-soluble parasite-specific protein released from parasite-infected erythrocytes. PfHRP-2 is produced throughout the parasite’s 48-h life cycle, with approximately 90% released at the moment of schizont rupture [18]. Quantitative measurement of plasma PfHRP-2 is a metric of the recent level of total circulating rings and sequestered trophozoite parasite biomass, related to parasite antigen release throughout the parasite life cycle, in contrast to quantification of only rings on a peripheral blood smear [19]. Semi-quantitative assessments of plasma PfHRP-2 concentrations in adults show a correlation with disease severity [20]. In African children presenting with febrile illness, plasma PfHRP-2 concentrations on admission predict mortality, and in those presenting with uncomplicated malaria, is predictive of progression to severe malaria [21–23]. In a study of Tanzanian children with uncomplicated or cerebral malaria, plasma PfHRP-2 was associated with malaria severity and mortality, though patients with CM were not defined by malaria retinopathy status [24]. Other patient cohorts have not identified a relationship between plasma PfHRP-2 and mortality in CM [21]. PfHRP-2 has recently been shown to be an important factor in CM pathogenesis. PfHRP-2 acts as a parasite virulence factor that activates the host innate immune system through an inflammasome-mediated pathway, causing redistribution of endothelial junctional proteins and increased BBB permeability [25]. Plasma PfHRP-2 does not represent brain sequestration in isolation, as sequestration in severe malaria involves multiple organ sites [6, 26]. This study therefore focuses on the measurement of PfHRP-2 in the CSF compartment. PfHRP-2 has previously been quantified in the CSF of patients with CM, though samples in this previous study were obtained without information on malaria retinopathy status [27]. Here, the authors report quantitative CSF PfHRP-2 levels in a cohort of retinopathy-positive CM children to determine whether there is a relationship between CSF PfHRP-2 and clinical, laboratory and radiographic features of interest.

## Methods

### Study population

A retrospective study was performed of children with CM between the ages of 6 months and 10 years admitted to the Queen Elizabeth Central Hospital Pediatric Research Ward (Blantyre, Malawi) between 2009 and 2013 meeting the inclusion criteria. Inclusion criteria were patients meeting World Health Organization (WHO) criteria for CM with findings of malarial retinopathy, who had a lumbar puncture (LP) performed on admission, with stored CSF and plasma samples available for testing. Patients undergoing traumatic taps [defined by CSF red blood cells (RBCs) > 10 cells/µL] were excluded from the study to avoid plasma pfHRP-2 contamination of CSF. All children received standard of clinical care while on the inpatient ward, including appropriate anti-malarial, antipyretic and anticonvulsant medications.

### Clinico-radiographic parameters

Demographic information was collected and human immunodeficiency (HIV) testing performed on admission on all patients whose guardians gave consent. Cases were identified as malarial retinopathy-positive based on at least one of the following changes in the optic fundus on indirect ophthalmoscopy examination, performed by an ophthalmologist with expertise in the diagnosis of malarial retinopathy: retinal haemorrhages, retinal whitening, and/or orange or white vessel discoloration. Patients with evidence of papilloedema on admission were excluded from the study as they did not undergo LP. Venous blood was drawn on admission for subsequent PfHRP-2 analysis, and finger-prick samples were analysed to determine parasite species and density, packed cell volume, blood glucose, and lactate concentrations. Additional laboratory parameters analysed on admission included white blood cell (WBC) count, platelet count, quantitative peripheral parasitaemia, CSF WBC count, and CSF protein. A sub-set of patients underwent brain MRI during their hospital admission. Ninety per cent of scans were performed within the first 24 h of hospitalization. Scanning was performed with a General Electric Signa Ovation Excite 0.35T Magnet (GE Healthcare, Milwaukee, WI, USA). Details of the scanning protocols used for the patients are found elsewhere [14]. Two radiologists (a neuroradiologist and a radiologist with fellowship training in neuroimaging), masked to the other’s readings, patient’s retinopathy status and clinical outcome interpreted MRI studies. Independent readings were performed using the NeuroInterp program, a searchable database based on a scoring system of brain MRI interpretation [28]. Using systems that require categorical or dichotomous assessments, radiologists grade T2 signal or diffusion weighted imaging (DWI) changes in various cranial structures, including (but not limited to) supratentorial white and grey matter, the posterior fossa, corpus callosum, and basal ganglia. Overall brain volume was scored based on the appearance of the cerebral hemispheres on axial T2 sequences. A scale from 1 to 8 was used; 1 and 2 represented some level of atrophy, 3 normal volume, 4 and 5 increasingly mild levels of increased volume, 6 obvious but moderate levels of increased brain volume, 7 significantly increased volume with diffuse sulcal and cisternal effacement but no evidence of herniation, and 8 sulcal and cisternal effacement with evidence of herniation. Volume scores of 7 and 8 were defined as severely increased brain volume prior to data analysis.

The primary clinical outcome of interest was death during hospitalization. Co-variables of interest included known laboratory markers of poor outcome in severe malaria including age, admission lactate, WBC count, and severely increased brain volume score on MRI.

### PfHRP-2 determination

Venous blood was drawn and CSF obtained at the time of admission. CSF and plasma were both stored at − 80 °C until the time of analysis. Aliquots of CSF or plasma were thawed and analysed in batches. Plasma or CSF were diluted at a ratio of either 1:100 or 1:500 in phosphate-buffered saline and tested as previously described [23].

### Statistical analysis

Patient demographic and clinical characteristics were summarized using appropriate descriptive statistics, e.g., counts and percentages, means and standard deviations (SDs), and medians and interquartile ranges (IQRs). Non-parametric analysis methods were used due to non-Gaussian distribution of data. Characteristics of patients who survived to discharge and those who died during hospitalization were compared using Fisher’s exact tests or Wilcoxon rank-sum tests. Wilcoxon signed-rank tests were used to determine the statistical differences between levels of PfHRP-2 in paired CSF and plasma samples. Spearman correlation was used to determine the relationship between CSF PfHRP-2 levels and MRI features of interest. Sensitivity, specificity and exact binomial 95% confidence intervals (CIs) for each variable of interest were calculated. Univariable logistic regression analysis examined clinical and radiographic characteristics with relationship to the level of PfHRP-2 in plasma, CSF or the CSF/plasma ratio. Multivariable logistic regression was performed to examine the association between potential predictors and the likelihood of death. Variables included in the multivariable logistic analysis were those determined a priori based on clinical relevance; age (6 month increments), abnormal WBC count on admission (> 10,000 WBC/µL), and abnormal admission lactate (lactate > 5 mmol/L). Odds ratios (ORs) and 95% CIs were used to quantify the strength of these associations. Analyses were performed using SAS version 9.3 (SAS Institute, Inc, Cary, NC, USA). All tests were two-sided and considered significant at p < 0.05.

## Results

### Patient characteristics

**Fig. 1:**
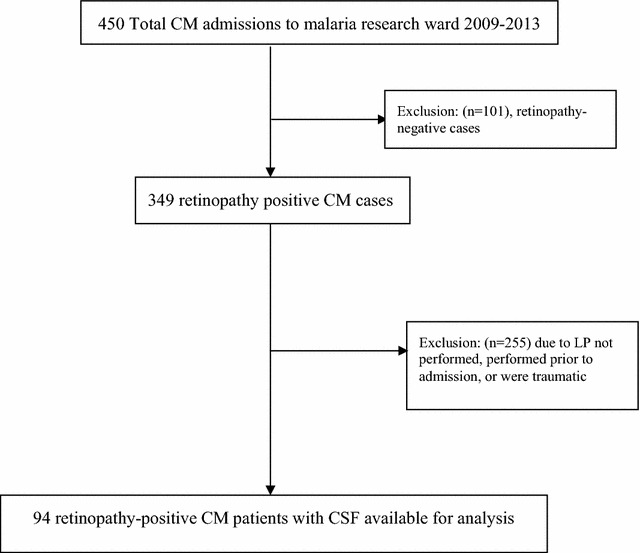
Flow-chart of inclusion and exclusion of patients in the study

There were 450 CM admissions to the malaria research ward during the period of 2009–2013, 349 (77.6%) of which had evidence of malarial retinopathy. Forty-nine patients were excluded due to traumatic LPs. LP was not performed in 80 individuals, due to papilloedema on ophthalmoscopy assessment or if the child was deemed too clinically unstable to undergo the procedure. In 126 patients, CSF samples were not available from patients who had an LP performed prior to admission to the malaria research ward (either on the paediatric inpatient ward or in the emergency department). Paired plasma and CSF samples from non-traumatic LPs were available in 94 retinopathy-positive CM patients (Fig. 1). Patients included in the study and those excluded did not differ statistically in clinical or laboratory parameters (Additional file 1: Table S1). Among the 94 retinopathy-positive patients, the median age was 44 (IQR 29–62) months, 53 patients (56.4%) were male, 6 (7%) were HIV infected, and 10 (11%) died during hospitalization (Table 1). Twenty-seven (29.3%) patients were hyperparasitaemic defined by parasite count > 250,000/µL, 55 (60%) patients had serum lactate > 5 mmol/L, 2 (2.1%) patients had serum glucose < 2.5 mmol/L, and 39 (46%) had platelet counts < 50,000/µL. Cellular and biochemical CSF findings were documented in 86 patients on admission with a median protein of 21 mg/mL with 14 (16%) patients having protein levels over 50 mg/mL and median CSF WBC of 0/mm with 13 (9%) having values > 5/mm^3^. Laboratory parameters stratified by outcome are shown in Table 2.

**Table 1:** Demographic and clinical characteristics

Characteristic	Retinopathy-positive CM (n = 94)
Age (months)	44 (29–62)
Male sex, n (%)	53 (56.4)
HIV infected, n (%)^a^	6 (7)
Glucose (mmol/L)	6.2 (4.6–7.3)
Haematocrit (%)^b^	20.5 (16.6–25.1)
White blood cell count (× 10^3^ cells/µL)^c^	8.5 (6.4–14.4)
Platelet count (× 10^3^ cells/µL)^d^	54 (31–80)
Lactate (mmol/L)^e^	7.0 (3.2–11.6)
Peripheral parasitaemia (× 10^3^ parasites/µL)^b^	83.7 (24.5–284.5)
Brain volume score > 6, n (%)^b^	13 (14)
In-hospital death, n (%)	10 (11)

Results presented are median (IQR) unless otherwise specified

^a^HIV testing in 85 patients

^b^n = 92

^c^n = 87

^d^n = 85

^e^n = 93

**Table 2:** Clinical and laboratory characteristics by survival

Parameter	Survived		Died		p value^a^
	# of patients	Median (IQR)	# of patients	Median (IQR)	
Age (months)	84	45 (28.5–61.5)	10	40 (33–66)	0.99
Glucose (mmol/L)	83	6.2 (4.6–7.3)	10	5.9 (4.5–10.1)	0.56
Haematocrit (%)	83	20.4 (16.1–24.6)	9	24.3 (17.7–27.6)	0.34
White blood cell count (× 10^3^ cells/µL)	78	8.3 (6.4–12.9)	9	11.0 (6.8–20.9)	0.17
Platelet count (× 10^3^ cells/µL)	76	54 (33–83)	9	31 (20–66)	0.23
Lactate (mmol/L)	82	5.9 (3.1–10.2)	10	12.6 (8.1–14.9)	0.005
Peripheral parasitaemia (× 10^3^ parasites/µL)	83	95.9 (25.0–283.9)	9	49.6 (22.0–554.2)	0.83
CSF PfHRP-2 (ng/mL)	84	19.6 (4.0–48.0)	10	54.6 (26.3–72.3)	0.04
Plasma PfHRP-2 (ng/mL)	84	9565 (3639–17,890)	10	15,597 (3880–19,172)	0.47

IQR, interquartile range

^a^p values are from Wilcoxon rank-sum tests

### Radiographic features

**Fig. 2:**
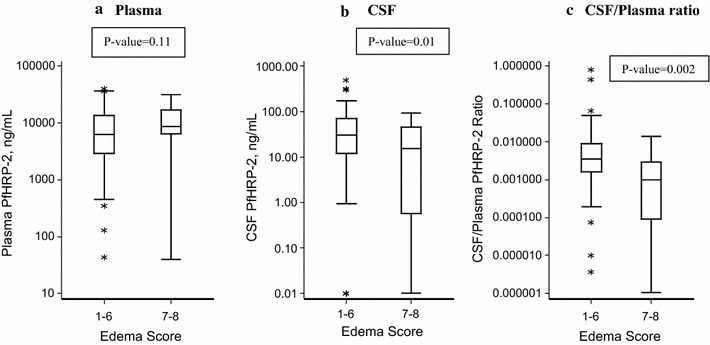
Comparison of PfHRP2 levels in **a** plasma, **b** CSF, and **c** the CSF/Plasma ratio in patients with severely increased brain volume scores versus those with mild-moderate increased brain volume. Line represents median value, box extends from first quartile to third quartile, whiskers extend to 5th and 95th percentile, and asterisks represent data points outside this range

Fifty-eight (62%) patients had brain MRIs performed. On univariable analysis, severely increased brain volume, defined by brain volume scores of 7 or 8, was associated with death (p = 0.048). Brain volume scores of 1–6 were significantly associated with higher CSF PfHRP-2 compared to scores of 7–8 (p = 0.01) (Fig. 2). CSF PfHRP-2 levels were significantly higher for patients with increased signal on diffusion weighted imaging in the globus pallidi (p = 0.02) and T2 signal abnormalities in the periventricular region (p = 0.03). These differences became non-significant with Bonferroni correction for multiple testing (Table 3).

**Table 3:** Differences in CSF PfHRP-2 and plasma PfHRP-2 levels by finding for 58 patients with brain magnetic resonance imaging

Radiographic finding	N (%) positive	Plasma PfHRP-2, median (IQR)			CSF PfHRP-2, median (IQR)		
		Positive finding	Negative finding	p value^a^	Positive finding	Negative finding	p value^a^
Frontal–occipital lobe T2 hyperintensity	6 (10)	37.9 (13.7–65.7)	24.3 (8.5–51.1)	0.72	5782 (2602–21,100)	12,906 (4820–22,569)	0.35
Periventricular T2 hyperintensity	25 (43)	15.2 (0.6–36.4)	31.6 (14.8–65.3)	0.03	11,000 (4258–17,510)	15,094 (5573–28,084)	0.17
Periventricular DWI abnormality	22 (38)	17.7 (8.8–33.3)	32.6 (8.1–64.8)	0.25	9951 (3631–19,172)	14,226 (5542–26,893)	0.27
Subcortical white matter T2 hyperintensity	39 (67)	24.7 (13.5–63.7)	30.1 (2.8–48.1)	0.90	11,000 (5294–21,100)	16,099 (3968–26,906)	0.80
Cortical T2 hyperintensity	47 (81)	27.9 (9.9–48.2)	14.2 (3.8–96.1)	0.77	13,358 (5294–26,879)	10,242 (3595–17,533)	0.20
Cortical DWI abnormality	6 (10)	5.0 (0–27.9)	27.7 (13.6–64.0)	0.06	14,754 (1678–28,084)	11,847 (5403–20,136)	0.69
Caudate T2 hyperintensity	41 (71)	29.1 (13.5–64.3)	15.2 (8.2–43.0)	0.31	13,358 (5862–26,879)	8147 (3968–17,533)	0.13
Caudate DWI abnormality	4 (7)	69.0 (18.5–212.0)	24.3 (8.8–48.2)	0.23	15,390 (11,047–26,981)	11,184 (4274–22,110)	0.51
Globus pallidi T2 hyperintensity	43 (74)	30.1 (13.5–65.3)	14.8 (6.2–35.1)	0.11	15,094 (5863–26,906)	5512 (3968–17,533	0.49
Globus pallidi DWI abnormality	13 (22)	65.3 (26.3–92.7)	19.2 (8.2–46.3)	0.02	15,094 (11,000–36,540)	10,470 (4258–21,100)	0.09
Putamen T2 hyperintensity	42 (72)	29.6 (13.5–64.3)	15.0 (5.5–39.0)	0.19	14,783 (5662–26,906)	6830 (2830–16,816)	0.04
Putamen DWI abnormality	7 (12)	63.7 (13.7–101.6)	23.8 (8.2–48.1)	0.11	13,358 (10,242–36,540)	11,128 (4258–22,110)	0.28
Thalamus T2 hyperintensity	31 (53)	26.3 (8.8–48.2)	19.2 (8.2–65.7)	0.99	13,358 (3902–26,906)	11,000 (5512–17,732)	0.61
Corpus callosum T2 hyperintensity	20 (34)	22.4 (13.7–44.6)	29.0 (2.8–64.3)	0.85	13,852 (5434–21,605)	11,847 (4258–26,906)	0.921
Corpus callosum DWI abnormality	19 (33)	24.7 (13.7–46.3)	27.9 (2.8–64.3)	0.86	17,462 (5573–23,028)	11,240 (3968–18,048)	0.49
Posterior fossa T2 hyperintensity	34 (59)	30.3 (13.5–64.3)	17.2 (4.5–47.9)	0.29	17,486 (5992–28,084)	8442 (4113–16,760)	0.02
Posterior fossa DWI abnormality	4 (7)	50.7 (4.4–207.6)	25.5 (9.9–48.2)	0.74	7526 (852–14,226)	11,847 (5294–23,028)	0.14

### Predictors of mortality

**Fig. 3:**
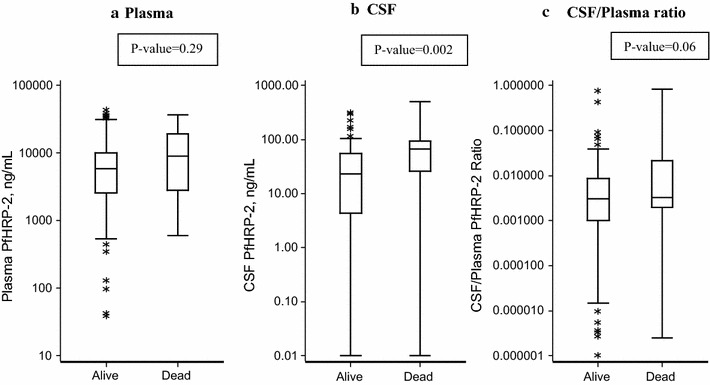
Comparison of PfHRP2 levels in **a** plasma, **b** CSF, and **c** the CSF/plasma ratio in patients who survived compared to those who died. Line represents median value, box extends from first quartile to third quartile, whiskers extend to 5th and 95th percentile, and asterisks represent data points outside this range

On univariable analysis, elevated lactate (p = 0.005) and CSF PfHRP-2 (p = 0.04) were significantly associated with death while other clinical and-laboratory parameters were not associated with mortality (Fig. 3, Table 2). After adjusting for age, abnormal WBC count on admission, and abnormal lactate, higher levels of CSF PfHRP-2 (OR 9.00, 95% CI 1.44–56.42) were associated with increased odds of in-hospital death while plasma PfHRP-2 (OR 2.05, 95% CI 0.45–9.35) and the CSF/plasma PfHRP-2 ratio (OR 1.67, 95% CI 0.35–7.88) were not associated with mortality (Table 4).

**Table 4:** Multivariable logistic regression analysis for plasma PfHRP-2, CSF PfHRP-2, and CSF/plasma PfHRP-2 ratio and mortality

Variable^a^	Plasma PfHRP-2 model		CSF PfHRP-2 model		CSF/plasma PfHRP-2 model	
	Odds ratio (95% CI)	p value	Odds ratio (95% CI)	p value	Odds ratio (95% CI)	p value
Age, 6 month increase	1.08 (0.92–1.26)	0.33	1.04 (0.88–1.24)	0.63	1.05 (0.90–1.23)	0.51
Abnormal WBC vs normal	3.29 (0.76–14.17)	0.11	6.61 (1.05–41.73)	0.04	2.94 (0.70–12.26)	0.14
Abnormal lactate vs normal	11.74 (0.76–181.61)	0.08	22.47 (1.07–470.35)	0.04	12.66 (0.82–194.18)	0.07
High plasma PfHRP-2 vs low	2.05 (0.45–9.35)	0.35	–		–	–
High CSF PfHRP-2 vs low	–	–	9.00 (1.44–56.42)	0.02	–	
High CSF/plasma PfHRP-2 vs low	–	–	–	–	1.67 (0.35–7.88)	0.52

*CI* confidence interval, *WBC* white blood count

^a^White blood cell count was coded abnormal if ≥ 10,000 cells/μL; lactate was coded abnormal if ≥ 5 mmol/L; PfHRP-2 measures were coded high if in the upper quartile (CSF > 65.309 ng/mL, ratio > 0.00945, plasma > 10,242 ng/mL) and low if less than the upper quartile. Similar results were obtained if these measures were continuous

## Discussion

In this study, assessment of the levels of PfHRP-2 was performed in the CSF and plasma in retinopathy-positive children with paediatric CM to determine whether PfHRP-2 is associated with clinical, laboratory and radiographic features during acute illness. Peripheral blood parasitaemia and plasma PfHRP-2 have often been used as a marker for disease severity, although not associated with mortality in this study [20–22, 24]. Measurements in the peripheral blood do not reflect CNS pathology in isolation, as sequestration occurs in multiple organs. In contrast, CSF PfHRP2 levels may reflect CNS specific sequestration [26]. Previous studies have suggested that BBB permeability may play a critical role in the underlying pathophysiology of paediatric CM [11, 29]. Increased permeability of the BBB may allow for pathophysiologic and immunologic communication between the blood stream and brain parenchyma, and most commonly occurs in parallel with disruption of the blood CSF barrier (BCSFB) [30]. Under steady state, horseradish peroxidase, a protein tracer with a similar molecular weight (40 kDa) to PfHRP-2 (37 kDa) does not cross the intact choroidal epithelium [31, 32]. The presence of any PfHRP-2 in the CSF is therefore indicative that the BCSFB has been compromised. In survivors, it is likely that the BCSFB is less leaky and therefore smaller amounts of PfHRP-2 are able to diffuse across the choroid plexus. As the BCSFB barrier becomes more permeable, PfHRP-2 is able to diffuse through the interepithelial tight junctions more easily and, therefore, it is hypothesized that elevated levels of CSF PfHRP-2 are seen in more severe cases of CM. Although these findings show the ratio of CSF/plasma PfHRP-2 was not associated with survival, this finding may be related to the limited ability of PfHRP-2 to enter the CSF space when patients develop severely increased intracranial pressure. Likewise, the decreased levels of CSF PfHRP-2 in patients with severely increased brain volume may reflect the hydrodynamics of decreased flow across the compromised BCSFB in these instances.

The biologic significance of increased PfHRP-2 in the CSF remains unclear. Many factors likely contribute to CM pathogenesis; the mechanisms that lead to death are incompletely understood. PfHRP-2, which is unique to *P. falciparum*, the most pathogenic of the human *Plasmodium* species, has proposed functions which include binding zinc and haem, mediating formation of haemozoin as well as immunomodulatory effects and perturbations of the coagulation cascade [33–35]. Recent studies have shown that the molecule has a direct effect on endothelial barrier function, with parasite strains lacking in the production of PfHRP-2 unable to lead to barrier destruction in the way that PfHRP-2 positive strains do. The barrier disruption is able to be recapitulated with either native or recombinant PfHRP-2 protein [25]. Further studies should be performed to examine the role of PfHRP-2 in vivo and its mechanisms for entry into the CSF space.

In this study, comparison of various clinical and radiographic features in relation to plasma and CSF PfHRP-2 and mortality was also performed. A previous study in Nigerian children with CM found that age under 3 years, abnormal breathing pattern, hypoglycaemia and leukocytosis were predictive of mortality [36]. In a large, multicentre, randomized, control trial, acidosis, cerebral involvement, renal impairment, and chronic illness were key independent predictors for a poor outcome in African children with severe malaria [37]. In this cohort, admission lactate was found to be predictive of mortality although no other clinical or laboratory parameters were found to be associated with mortality. Although this could be attributable to this study’s relatively small study size, it could also be due to inclusion of only retinopathy positive patients. This would serve to ‘homogenize’ the patients, eliminating retinopathy-negative patients with coma aetiology other than severe malaria. This misclassification of retinopathy-negative patients would serve to accentuate clinical or laboratory variables that are unique to malarial versus non-malarial causes of death. Elevated CSF PfHRP-2 was associated with lower brain volume score, which is surprising as a prior study in retinopathy-positive CM cases in Malawi showed that higher brain volume score is associated with mortality [17]. The lack of relationship of CSF PfHRP2 with higher brain volume scores is likely related to the significant number of patients excluded from this study because they had evidence of papilloedema, and did not undergo LP. There were 16 additional children with oedema scores of 7 or 8 evaluated in the prior study who had evidence of papilloedema, and did not undergo LP. There were several radiologic findings that were associated with higher CSF PfHRP-2 findings, although the association became non-significant when adjusting for the number of statistical evaluations performed. CM presents with a widely heterogeneous radiological pattern [14]. Unlike other neurologic syndromes there is no single radiographic finding that will define CM. It is unclear whether this diverse pattern of findings is related to timing of the MRI scan relative to natural history of the disease, or whether there are distinct pathophysiologic mechanisms within the single disease classification. Given this variability in MRI findings, as well as the highly variable levels of CSF PfHRP-2 it is not surprising that correlations have not reached statistical significance; further studies with greater sample size may help to clarify the relationship between damage to particular brain regions (as determined by MRI) and egress of PfHRP-2 to the CSF space.

There are several strengths of the study. PfHRP-2 was tested in a large cohort of paediatric CM patients, with extensive clinical, laboratory and radiographic characterization. Examination of retinopathy-positive CM cases, which represent a cohort of true CM cases, with eye examinations documented by trained ophthalmologists was performed. Paired plasma and CSF samples allowed for direct comparisons of plasma and CSF pfHRP-2. Additionally, detailed clinical, laboratory and radiographic interpretation was available in a large group of paediatric patients, which has not been previously described with relation to CSF or plasma biomarkers.

There were several limitations to the study. The sample excluded subjects with papilloedema, as they did not undergo LP. However, even after excluding these cases, a relationship between CSF PfHRP-2 and outcome was identified. Also, many cases were excluded because children had an LP prior to admission to the malaria research ward, resulting in the CSF sample being unavailable for PfHRP-2 analysis. A selected group who had MRI scans was examined and results related to neuroimaging findings may be influenced by the selected patients, who were able to undergo neuroimaging. There was no evaluation of PfHRP-2 with respect to longitudinal neurological outcome as these data were not available in the patient cohort, though this would be valuable to investigate as children with CM often have neurological sequelae. There was no evaluation of plasma and CSF PfHRP-2 in parasitaemic children with no neurological symptoms, as doing an LP in that setting would be unethical. Based on previous observations though, it may be important to quantify CSF PfHRP-2 in those children with less severe malaria infection and those with severe, non-cerebral malaria, including those who are comatose though do not have findings of malarial retinopathy. Additionally, the focus here was on CSF PfHRP-2 but examining other markers of BCSFB and BBB function would be insightful.

## Conclusion

The study results demonstrate that there is a significant relationship between CSF PfHRP-2 and mortality in paediatric cases of retinopathy-positive CM. Previous studies have found that plasma PfHRP-2 is associated with mortality in patients with severe malaria [21, 24]. This study differs in that this sample was defined by clinical evidence of retinal and cerebral neurovascular pathology, rather than the broader standard clinical case definition of CM, which may include patients in coma with alternate aetiologies from CM in high burden malaria settings. Within this cohort of children with CM, plasma PfHRP-2 did not show an association with mortality, probably because a very select group of patients with brain involvement was examined, all of which had markedly raised plasma PfHRP-2 levels. The association of CSF levels in the absence of a correlation with plasma PfHRP-2 suggests that blood–CSF dynamics may be altered in CM and may play an important role in CM pathobiology. Further studies are needed to define the role of PfHRP-2 in CM pathogenesis, and to further elucidate the degree of BBB and BCSFB disruption in CM. Additionally, given the varied findings on brain MRI in CM, further studies are needed to establish whether there is a relationship between structural abnormalities seen in CM and disease biomarkers.

## Additional file

**Additional file 1: Table S1.** Clinical and laboratory characteristics of patients included in and excluded from the study.

## References

[1] WHO (2015). World malaria report.

[2] World Health Organization (2000). Severe falciparum malaria. World Health Organization, communicable diseases cluster. Trans R Soc Trop Med Hyg.

[3] Beare NA, Taylor TE, Harding SP, Lewallen S, Molyneux ME (2006). Malarial retinopathy: a newly established diagnostic sign in severe malaria. Am J Trop Med Hyg.

[4] Lewallen S, Taylor TE, Molyneux ME, Wills BA, Courtright P (1993). Ocular fundus findings in Malawian children with cerebral malaria. Ophthalmology.

[5] Lewallen S, Bronzan R, Beare N, Harding S, Molyneux M, Taylor T (2008). Using malarial retinopathy to improve the classification of children with cerebral malaria. Trans R Soc Trop Med Hyg.

[6] Seydel KB, Milner DA, Kamiza SB, Molyneux ME, Taylor TE (2006). The distribution and intensity of parasite sequestration in comatose Malawian children. J Infect Dis.

[7] Taylor T, Fu W, Carr R, Whitten RO, Mueller JS, Fosiko NG (2004). Differentiating the pathologies of cerebral malaria by postmortem parasite counts. Nat Med.

[8] Ponsford MJ, Medana IM, Prapansilp P, Hien TT, Lee SJ, Dondorp AM (2012). Sequestration and microvascular congestion are associated with coma in human cerebral malaria. J Infect Dis.

[9] van der Heyde HC, Nolan J, Combes V, Gramaglia I, Grau GE (2006). A unified hypothesis for the genesis of cerebral malaria: sequestration, inflammation and hemostasis leading to microcirculatory dysfunction. Trends Parasitol.

[10] Miller LH, Baruch DI, Marsh K, Doumbo OK (2002). The pathogenic basis of malaria. Nature.

[11] Dorovini-Zis K, Schmidt K, Huynh H, Fu W, Whittne RO, Milner D (2011). The neuropathology of fatal cerebral malaria in Malawian children. Am J Pathol.

[12] MacCormick IJ, Beare NA, Taylor TE, Barrera V, White VA, Hiscott P (2014). Cerebral malaria in children: using the retina to study the brain. Brain.

[13] Potchen M, Birbeck G, Demarco J, Kampondeni SD, Beare N, Molyneux M (2009). Neuroimaging findings in children with retinopathy-confirmed cerebral malaria. Eur J Radiol.

[14] Potchen MJ, Kampondeni SD, Seydel KB, Birbreck GL, Hammond CA, Bradley WG (2012). Acute brain MRI findings in 120 Malawian children with cerebral malaria: new insights into an ancient disease. AJNR Am J Neuroradiol.

[15] Newton CR, Peshu N, Kendall B, Kirkham FJ, Sowunmi A, Waruiru C (1994). Brain swelling and ischaemia in Kenyans with cerebral malaria. Arch Dis Child.

[16] Gamanagatti S, Kandpal H (2006). MR imaging of cerebral malaria in a child. Eur J Radiol.

[17] Seydel KB, Kampondeni SD, Valim C, Potchen MJ, Milner DA, Muwalo FW (2015). Brain swelling and death in children with cerebral malaria. N Engl J Med.

[18] Desakorn V, Dondorp AM, Silamut K, Pongtavornpinyo W, Sahassananda D, Chotivanich K (2005). Stage-dependent production and release of histidine-rich protein 2 by *Plasmodium falciparum*. Trans R Soc Trop Med Hyg.

[19] Dondorp AM, Desakorn V, Pongtavornpinyo W, Sahassananda D, Silamut K, Chotivanich K (2005). Estimation of the total parasite biomass in acute falciparum malaria from plasma PfHRP2. PLoS Med.

[20] Desakorn V, Silamut K, Angus B, Sahassananda D, Chotivanich K, Suntharasamai P (1997). Semi-quantitative measurement of *Plasmodium falciparum* antigen PfHRP2 in blood and plasma. Trans R Soc Trop Med Hyg.

[21] Hendriksen IC, Mwanga-Amumpaire J, von Seidlein L, Mtove G, White LJ, Olaosebikan R (2012). Diagnosing severe falciparum malaria in parasitaemic African children: a prospective evaluation of plasma PfHRP2 measurement. PLoS Med.

[22] Hendriksen IC, White LJ, Veenemans J, Mtove G, Woodrow C, Amos B (2013). Defining falciparum-malaria-attributable severe febrile illness in moderate-to-high transmission settings on the basis of plasma PfHRP2 concentration. J Infect Dis.

[23] Fox LL, Taylor TE, Pensulo P, Liomba A, Mpakiza A, Varela A (2013). Histidine-rich protein 2 plasma levels predict progression to cerebral malaria in Malawian children with *Plasmodium falciparum* infection. J Infect Dis.

[24] Rubach MP, Mukemba J, Florence S, John B, Crookston B, Loansri BK (2012). Plasma *Plasmodium falciparum* histidine-rich protein-2 concentrations are associated with malaria severity and mortality in Tanzanian children. PLoS ONE.

[25] Pal P, Daniels BP, Oskman A, Diamond MS, Klein RS, Goldberg DE (2016). *Plasmodium falciparum* histidine-rich protein ii compromises brain endothelial barriers and may promote cerebral malaria pathogenesis. MBio.

[26] Milner DA, Lee JJ, Frantzreb C, Whitten RO, Kamiza S, Carr RA (2015). Quantitative assessment of multiorgan sequestration of parasites in fatal pediatric cerebral malaria. J Infect Dis.

[27] Mikita K, Thakur K, Anstey NM, Piera KA, Pardo CA, Weinberg JB (2014). Quantification of *Plasmodium falciparum* histidine-rich protein-2 in cerebrospinal spinal fluid from cerebral malaria patients. Am J Trop Med Hyg.

[28] Potchen MJ, Kampondeni SD, Ibrahim K, Bonner J, Seydel KB, Taylor TE (2013). NeuroInterp: a method for facilitating neuroimaging research on cerebral malaria. Neurology.

[29] Abbott NJ, Ronnback L, Hansson E (2006). Astrocyte–endothelial interactions at the blood–brain barrier. Nat Rev Neurosci.

[30] Johanson C, Nag S (2011). The blood–cerebrospinal fluid barrier: structure and functional significance. The blood–brain and other neural barriers: reviews and protocols.

[31] Rapoport SI, Pettigrew KD (1979). A heterogenous, pore-vesicle membrane model for protein transfer from blood to cerebrospinal fluid at the choroid plexus. Microvasc Res.

[32] Feder N (1971). Microperoxidase. An ultrastructural tracer of low molecular weight. J Cell Biol.

[33] Schneider EL, Marletta MA (2005). Heme binding to the histidine-rich protein II from *Plasmodium falciparum*. Biochemistry.

[34] Sullivan DJ, Gluzman IY, Goldberg DE (1996). Plasmodium hemozoin formation mediated by histidine-rich proteins. Science.

[35] Das P, Grewal JS, Chauhan VS (2006). Interaction of *Plasmodium falciparum* histidine-rich protein II with human lymphocytes leads to suppression of proliferation, IFN-gamma release, and CD69 expression. Parasitol Res.

[36] Oluwayemi OI, Brown BJ, Oyedeji OA, Adegoke SA, Adebami OJ, Oyedeji GA (2013). Clinical and laboratory predictors of outcome in cerebral malaria in suburban Nigeria. J Infect Dev Ctries.

[37] von Seidlein L, Olaosebikan R, Hendriksen IC, Lee SJ, Adedoyin OT, Agbenyega T (2012). Predicting the clinical outcome of severe falciparum malaria in african children: findings from a large randomized trial. Clin Infect Dis.

