# Supplementary material for: Cerebrospinal fluid Plasmodium falciparum histidine-rich protein-2 in pediatric cerebral malaria
Source: Malar J. 2018 Mar 23;17:125. doi: 10.1186/s12936-018-2272-y (PMC5865338; doi:10.1186/s12936-018-2272-y)
Supplement: Supplementary file 1 — Additional file 1: Table S1. Clinical and laboratory characteristics of patients included in and excluded from the study. [file 12936_2018_2272_MOESM1_ESM.docx]

**Table S1: Clinical and laboratory characteristics of patients included in and excluded from the study**

| Characteristic | Included Patients (n=94) | Excluded Patients (n=255) | P-value^a^ |
| --- | --- | --- | --- |
| Age (months) | 44 (29-62) | 44 (30-67) | 0.93 |
| Male sex, n (%) | 53 (56.4) | 121 (47.5) | 0.15 |
| Hypoglycemic, n (%) | 2 (2.1) | 5 (2.0)^b^ | 1.0 |
| Lactate (mmol/L) | 6.7 (3.2 – 11.6)^b^ | 5.8 (3.1 – 10.4)^c^ | 0.35 |
| Hematocrit (%) | 20.5 (16.6 – 25.1)^b^ | 20.4 (17.4 – 25.1)^d^ | 0.76 |
| White blood cell count (cells/µL) | 8.5 (6.4 – 14.4)^e^ | 9.1 (6.7 – 13.8)^f^ | 0.45 |
| Platelet count (cells/µL) | 54 (31 – 80)^g^ | 53.0 (30.9 – 86.0)^h^ | 0.44 |
| Peripheral parasitemia  (x10^3^ parasites/µl) | 83.7 (24.2 – 284.5)^i^ | 71.3 (12.7 – 278.5)^c^ | 0.44 |
| HIV infected, n (%) | 6 (7.1)^g^ | 34 (14.3)^j^ | 0.09 |
| In-hospital death, n (%) | 10 (10.6) | 44 (17.3) | 0.14 |

Results presented are median (interquartile range) unless otherwise specified.

^a^ P-value from Wilcoxon rank-sum test or Fisher’s exact test.

^b^ 1 patient missing data

^c^ 6 patients missing data

^d^ 4 patients missing data

^e^ 7 patients missing data

^f^ 12 patients missing data

^g^ 9 patients missing data

^h^ 17 patients missing data

^i^ 2 patients missing data

^j^ 19 patients missing data
